# High-Level Rovibrational Calculations on Ketenimine

**DOI:** 10.3389/fchem.2020.623641

**Published:** 2021-01-06

**Authors:** Martin Tschöpe, Benjamin Schröder, Sebastian Erfort, Guntram Rauhut

**Affiliations:** Institute for Theoretical Chemistry, University of Stuttgart, Stuttgart, Germany

**Keywords:** ketenimine, *ab initio* calculations, Fermi resonances, rotational spectrum, VSCF/VCI theory, rovibrational calculations

## Abstract

From an astrochemical point of view ketenimine (CH_2_CNH) is a complex organic molecule (COM) and therefore likely to be a building block for biologically relevant molecules. Since it has been detected in the star-forming region Sagittarius B2(N), it is of high relevance in this field. Although experimental data are available for certain bands, for some energy ranges such as above 1200 cm^−1^ reliable data virtually do not exist. In addition, high-level *ab initio* calculations are neither reported for ketenimine nor for one of its deuterated isotopologues. In this paper, we provide for the first time data from accurate quantum chemical calculations and a thorough analysis of the full rovibrational spectrum. Based on high-level potential energy surfaces obtained from explicitly correlated coupled-cluster calculations including up to 4-mode coupling terms, the (ro)vibrational spectrum of ketenimine has been studied in detail by variational calculations relying on rovibrational configuration interaction (RVCI) theory. Strong Fermi resonances were found for all isotopologues. Rovibrational infrared intensities have been obtained from dipole moment surfaces determined from the distinguishable cluster approximation. A comparison of the spectra of the CH_2_CNH molecule with experimental data validates our results, but also reveals new insight about the system, which shows very strong Coriolis coupling effects.

## 1. Introduction

More than 200 molecules have been detected in the interstellar medium (ISM) or circumstellar shells (Müller et al., 2001, 2005; Endres et al., 2016; McGuire, 2018) presenting a chemical variety from rather stable to highly reactive species such as radicals, carbenes, and molecular ions. In a similar way, the size measured by the number of atoms varies substantially from simple diatomics (e.g., CO, CN, and OH; Weinreb et al., 1963; Jefferts et al., 1970; Wilson et al., 1970), to carbon-chain molecular species like cyanopolyynes (HC_*n*_N; Ohishi and Kaifu, 1998) and simple organic molecules like methanol (CH_3_OH; Ball et al., 1970), up to still larger compounds like polycyclic aromatic hydrocarbons (PAHs; Allamandola et al., 1989) and fullerenes (C_60_; Cami et al., 2010). Within the context of astrochemistry molecules with 6 or more atoms are usually referred to as complex molecules and when carbon is present also as complex *organic* molecules (COMs; Herbst and van Dishoeck, 2009). Such compounds are thought to be important building blocks for biologically relevant molecules (Woon, 2002; Theule et al., 2011; Ohishi, 2019) and accordingly, much attention has been paid to the study of formation pathways for COMs (Herbst and van Dishoeck, 2009; Öberg, 2016, and references therein).

Examples of such COMs are the class of imines (R-C=NH). They have been shown to be important intermediates for the hydrogenation of CN moieties (Theule et al., 2011; Krim et al., 2019). Recently, formation of imines has been reported for radiative-processing of ices. Vasconcelos et al. investigated the products from ion irradiation of N_2_-CH_4_ ice mixtures by *in-situ* Fourier transform infrared spectroscopy (FTIR) and, among others, methyleneimine was identified (Vasconcelos et al., 2020). In a similar fashion, Carvalho and Pilling (2020) detected ketenimine by FTIR spectroscopy upon irradiation of acetonitrile ice with X-rays (6–2000 eV). Ketenimine (H_2_C=C=NH) is one of only 4 imines which have been conclusively identified to be present in the ISM (Godfrey et al., 1973; Kawaguchi et al., 1992; Lovas et al., 2006; Zaleski et al., 2013). Using the 100 m Green Bank Telescope Lovas and coworkers observed three rotational transitions of ketenimine toward the star-forming region Sagittarius B2(N) (Lovas et al., 2006). It is known that temperatures in Sagittarius B2(N) vary between 40 K in the envelope and 300 K in the hot dense core (Martín-Pintado et al., 1996). Therefore, it could be possible that not only the rotational spectrum of ketenimine can be detected with radio telescopes, but also the rovibrational spectrum due to IR spectroscopy. Since Sagittarius B2(N) is a star forming region, the question arises whether ketenimine can be found in protoplanetary disks or even exoplanet atmospheres. Considering the recent successes in this field (Charbonneau et al., 2002; Mandell et al., 2013; Gandhi et al., 2020) as well as the upcoming space telescopes James Webb Space Telescope (JWST) and Atmospheric Remote-sensing Infrared Exoplanet Large-survey (ARIEL) with high sensitivity in this spectral range this is a feasible aim.

Given its importance as the simplest member of a larger class of chemically interesting molecules (Alajarin et al., 2012), ketenimine has been subject to a number of experimental spectroscopic investigations which revealed a complex rovibrational spectrum. The first spectroscopic investigation of ketenimine was reported by Jacox and Milligan (1963). Infrared transitions of the transient species were tentatively assigned following the reaction of NH with acetylene in argon matrix. The assignment was later confirmed and extended by Jacox (1979) in an argon matrix-isolation study of the photoisomerization of acetonitrile.

A gas phase rotational spectrum of ketenimine was obtained by Rodler and coworkers using microwave spectroscopy (Rodler et al., 1984). Ground-state rotational parameters of *A*_0_ = 201443.69, *B*_0_ = 9663.138, and *C*_0_ = 9470.127 MHz were determined from a fit to Watson's *S*-reduced rotational Hamiltonian (Watson, 1977) in the I^r^ representation. The latter parameters show that ketenimine is a near-prolate asymmetric top (asymmetry parameter κ = −0.998). Measurements of Stark-splittings (Rodler et al., 1984) yielded the ground state dipole moments ${\;}^{a}\mu_{0}=\text{0.431(1)}\;\text{D}$ and ${\;}^{c}\mu_{0}=\text{1.371(6)}\;\text{D}$. Rodler et al. later carried out a high-resolution analysis in the 4–7 GHz region for the main as well as the ND isotopologue revealing small splittings in the former case, due to the imino inversion (Rodler et al., 1986). The latest study of the vibrational ground state rotational spectrum of ketenimine was performed by Degli Esposti at submillimeter wavelength (Degli Esposti et al., 2014). In total, 297 line frequencies were analyzed yielding spectroscopic parameters that allow for the accurate prediction of rotational transitions up to 1 THz.

The rovibrational spectrum of ketenimine has been studied by both, in low-resolution (August, 1986) as well as high-resolution (Ito et al., 1990; Ito and Nakanaga, 2010; Bane et al., 2011a,b,c). A gas phase spectrum of the ${\tilde{\nu }}_{3}=2044$ cm^−1^ CCN-stretching vibration has been obtained by Ito et al. (1990) using FTIR spectroscopy. Analysis of the spectrum revealed a complicated structure due to several Coriolis-type interactions, which could only be analyzed approximately due to missing information on the perturbing states. Almost 20 years later Ito and Nakanaga reported the observation of the CNH bending rovibrational spectrum around ${\tilde{\nu }}_{6}=\text{1000}\;\text{cm}\text{-1}$ using FTIR spectroscopy. Again, strong Coriolis perturbations precluded a detailed analysis of the ν_6_ state and only effective spectroscopic parameters for individual *K*_*a*_ sub-bands were obtained. The latter values allowed the ν_10_ (CH_2_ rocking) and ν_11_ (torsion) vibrations to be identified as likely perturbers, based on their large contribution to the vibration-rotation interaction constant $\alpha_{6}^{A}$.

In a series of articles Bane and coworkers presented a thorough experimental analysis of the low lying fundamental bands of ketenimine (Bane et al., 2011a,b,c). The observed bands encompass the out-of-plane and in-plane CCN bending vibrations around ${\tilde{\nu }}_{12}=409$ and ${\tilde{\nu }}_{8}=\text{466}\;\text{cm}\text{-1}$ (Bane et al., 2011c), respectively, the CH_2_ wagging mode (${\tilde{\nu }}_{7}=\text{693}\;\text{cm}\text{-1}$; Bane et al., 2011b) and the CH_2_ rocking mode (${\tilde{\nu }}_{10}=\text{983}\;\text{cm}\text{-1}$) as well as the strong CNH bending mode ν_6_ (Bane et al., 2011a). Following the assignment of more than 6,000 rovibrational transitions and fitting of the spectrum, an intricate system of Coriolis-coupled states was revealed whereby all 5 observed states are coupled via Coriolis-coupling either directly (e.g., *a*-axis Coriolis coupled ν_12_ & ν_8_) or indirectly. The analysis required the inclusion of unobserved “dark states” 2ν_8_, ν_8_ + ν_12_, and 2ν_12_ which are also expected to be strongly Coriolis-coupled amongst themselves. While the global fit to Watson's *S*-reduced Hamiltonian (*I*^*r*^) reproduced the observed rovibrational transition frequencies, Bane et al. noted that the torsion fundamental ν_11_ around 880 cm-1 probably also adds to the complex rovibrational coupling but considered inclusion of this interaction intractable.

Theoretical work on the rotational and rovibrational spectroscopy of ketenimine is rather scarce and either based on limited *ab initio* methods (Kaneti and Nguyen, 1982; Brown et al., 1985) or has been done only in support of dedicated experimental investigations (Ito et al., 1990; Ito and Nakanaga, 2010; Bane et al., 2011c). In the latter case, the work of Bane and coworkers (Bane et al., 2011c) provided the previously most accurate predictions of the fundamental frequencies with a root-mean-squared deviation of 11 cm-1. The results were obtained from B3LYP/cc-pVTZ harmonic frequencies which were uniformly scaled by a factor of 0.965. Given its possible importance in astrochemical reaction networks and the strong rovibrational couplings a more in-depth look at the rotational and rovibrational spectroscopy of ketenimine appears desirable.

Recently, some of us reported on the implementation of a new program for variational rovibrational calculations within the Molpro package of *ab initio* programs (Erfort et al., 2020a). The approach combines the well established Molpro capabilities (Werner et al., 2020) of obtaining multidimensional potential energy and dipole moment surfaces, comprehensive symmetry information and the accurate determination of vibrational wave functions with efficient calculation of partition functions, rovibrational transition frequencies, and transition dipole matrix elements in an almost black-box manner. Within this study here, we report about high-level *ab initio* calculations based on anharmonic potential energy surfaces obtained from explicitly correlated coupled-cluster theory, which allows for a detailed analysis of the (ro)vibrational spectra of the title compound. Compared to previous work the rovibrational calculations have been extended by pure rotational spectra, which is a newly implemented feature in Molpro.

## 2. Computational Details

Geometries, harmonic frequencies and normal coordinates of ketenimine (X^1^A′) and its C_*s*_ symmetric isotopologues were computed at the level of frozen-core explicitly correlated coupled-cluster theory, CCSD(T)-F12b, in combination with a basis set of triple-ζ quality, i.e., cc-pVTZ-F12 (Adler et al., 2007). Hartree-Fock energies were corrected by addition of the complementary auxiliary basis set singles correction (CABS) (Knizia and Werner, 2008).

*n*-mode expansions of the potential energy surface (PES) and the dipole moment surface (DMS) being truncated after 4th order were used in all calculations (Ziegler and Rauhut, 2018). A multi-level scheme has been employed throughout (Pflüger et al., 2005; Yagi et al., 2007), in which the 1D and 2D terms of the PES were computed at the CCSD(T)-F12b/cc-pVTZ-F12 level, while the explicitly correlated distinguishable clusters approach, DCSD-F12b, in combination with a smaller cc-pVDZ-F12 basis was used for the 3D and 4D terms. The 1D and 2D terms of the DMS were computed at the conventional DCSD/cc-pVTZ-F12 level and the 3D and 4D terms at the DCSD/cc-pVDZ-F12 level (Kats and Manby, 2013; Kats et al., 2015). In total about 170,000 *ab initio* points were used for representing the surfaces. Efficient Kronecker product fitting was employed to transform this grid representation into an analytical one consisting of 10 local B-splines per dimension (Ziegler and Rauhut, 2016).

Vibrational self-consistent field theory (VSCF) has been used to determine one-mode wavefunctions (modals) based on the Watson Hamiltonian (Watson, 1968). Vibrational angular momentum terms (VAM) were not included within the variational determination of the modals, but were added *a posteriori* to the state energies (Neff et al., 2011). A mode-dependent basis of 20 distributed Gaussians has been used throughout for representing the modals. Subsequent state-specific configuration-selective vibrational configuration interaction calculations (VCI) were used for calculating accurate state energies (Neff and Rauhut, 2009). The correlation space contained single to 6-tuple excitations up to the 8th root per mode and a maximum sum of quantum numbers of 15. This resulted in about 4·10^6^ Hartree products (configurations) per irreducible representation. These calculations included VAM terms based on a constant ***μ***-tensor. Eigenvalues were determined with our residuum based eigenvalue solver (RACE) (Petrenko and Rauhut, 2017).

Within the calculation of the rovibrational spectra we also use the Watson-operator (Watson, 1968)

(1) $$\begin{array}{l} H_{\text{Watson}}=\frac{1}{2}\underset{\alpha \beta }{\sum }J_{\alpha }\mu_{\alpha \beta }J_{\beta }-\frac{1}{2}\underset{\alpha \beta }{\sum }\left(J_{\alpha }\mu_{\alpha \beta }\pi_{\beta }+\pi_{\alpha }\mu_{\alpha \beta }J_{\beta }\right)+H_{\text{Vib}}, \end{array}$$

where *J*_α_ denotes the total angular momentum operator, π_α_ the vibrational angular momentum operator and μ_αβ_ refers to an element of the inverse effective moment of inertia tensor. The summations over α and β run over the three molecule fixed Cartesian space coordinates. The first term in Equation (1) gives the kinetic energy of rotational motion and the second term couples rotation and vibration and is referred to as Coriolis term. All other terms of the Watson Hamiltonian are purely vibrational operators and are thus summarized in the term denoted *H*_Vib_. Within rovibrational configuration interaction (RVCI) theory the rovibrational wave functions are expanded in terms of products of VCI wave functions and rotational basis functions (Erfort et al., 2020a,b). The latter can be either primitive symmetric top eigenfunctions or Wang combinations of symmetric top functions (Wang, 1929; Špirko et al., 1985).

In the following, we will distinguish between rotational configuration interaction (RCI) and rovibrational configuration interaction (RVCI). RCI is an approximation, in which no rovibrational interaction between different vibrational states is considered. This corresponds to neglecting the second term, see Equation (1), as well as all terms off-diagonal in the vibrational quantum numbers, arising from the 1D and higher order expansion of the ***μ***-tensor (centrifugal distortion). Since every RCI-matrix is thus constructed for a single VCI wave function, the vibrational state identity can be trivially assigned for every rovibrational state. In contrast, within RVCI all rovibrational interactions are considered. As a consequence the only “good” quantum number is the angular momentum quantum number *J* and the parity of the rovibrational state. In this sense, RVCI yields the physically meaningful results. However, we found that a comparison of RVCI with the RCI results is helpful to understand and visualize both the effects of Coriolis interaction and intensity borrowing mechanisms in general. Again, it shall be noted that for comparison with experiments only RVCI results should be used.

The rovibrational intensities are calculated according to

(2) $$\begin{array}{l} I=\frac{2\pi^{2}}{3}\frac{N_{\text{A}}}{\epsilon_{0}h^{2}c^{2}}\frac{e^{-E^{\prime \prime }/k_{\text{B}}T}\left(1-e^{-\left(E^{\prime }-E^{\prime \prime }\right)/k_{\text{B}}T}\right)}{Q\left(T\right)}\left(E^{\prime }-E^{\prime \prime }\right)R^{2}. \end{array}$$

In Equation (2), the first two prefactors contain only constants. The next factor corresponds to the thermal distribution function, with the temperature *T*, Boltzmann constant *k*_B_, the energy of the lower state *E*″ and the upper state *E*′ as well as the temperature dependent partition function *Q*(*T*). For the latter, we use the separability approximation *Q* = *Q*_vib_*Q*_rot_ for several reasons. First, we are investigating a relatively low temperature regime up to 300 K, where the partition function converges quickly with increasing excitation, such that errors in energies for high-lying states have little influence. Second, we have shown in our previous work (Erfort et al., 2020a), that for H_2_CO and H_2_CS the differences between experimental *Q*(*T*) values and theoretically approximated *Q*_vib_*Q*_rot_ values for the partition function are lower than 2 % up to 300 K. In addition to that, the partition function is the same global factor for every transition and since we are primarily interested in relative intensities rather than in absolute intensities, it is therefore not crucial for us. The last two factors in Equation (2) correspond to the frequency of the transition (*E*′ − *E*″) and the squared transition moment *R*^2^. The calculation of the latter within RVCI has been outlined previously (Erfort et al., 2020a). For ketenimine all nuclear spin statistical weights show the values of 24.

Further approximations are used to limit the calculation times. First of all, we are not considering hot bands. Due to relatively low temperatures and absence of fundamentals with particularly low energies, these bands have fairly low intensities and are mainly hidden behind significantly more intense transitions arising from the vibrational ground state. This is supported by a comparison to the results of Bane and coworkers (Bane et al., 2011c). Moreover, the inverse effective moment of inertia tensor ***μ*** is expanded to the 0th order for the RVCI calculation. Within these computations all fundamental bands, seven combination bands (ν_3_ + ν_5_, ν_3_ + ν_6_, ν_5_ + ν_6_, ν_6_ + ν_10_, ν_7_ + ν_8_, ν_7_ + ν_12_, and ν_8_ + ν_12_) and seven overtones (2ν_6_, 2ν_7_, 2ν_8_, 2ν_10_, 2ν_12_, 3ν_8_, and 3ν_12_) were simultaneously considered, giving in total *N*_vib_ = 27 vibrational states (including the ground state). As a convergence check we performed a calculation with angular momentum quantum number of *J* ≤ 70 and one with *J* ≤ 100. The VCI calculations were performed in parallel using 9 cores, with a total computational time of 100 h. The required memory for the subsequent serial RVCI treatment is less than 40 GB. As an example, the RVCI matrix for *J* = 70, with *N*_vib_ = 27 vibrational states is of size (2*J* + 1)*N*_vib_ = 3, 807. Although, this is relatively small in comparison to other rovibrational software, the results are nevertheless very accurate. A possible reason for this lies in the very accurate and compact vibrational basis, in the form of VCI wavefunctions. Computational timings on a single CPU core were about 83 min for RVCI energies and about 14 h for RVCI intensities for *J* ≤ 70. For the same upper bound of *J* there were 3.81 × 10^7^ transitions considered and about 1.41 × 10^6^ of them where found to be significant. For *T* = 300 K the partition function is converged to *Q*(*T*) = 2.05 × 10^5^ at *J* = 71.

## 3. Results and Discussion

### 3.1. Geometrical Parameters, Rotational Constants, and Dipole Moments

Geometrical parameters of the main ketenimine isotopologue as well as two deuterated variants have been calculated and are provided in Table 1. The parameters obtained from the Born-Oppenheimer equilibrium geometry are denoted **r**_e_. The only experimental geometrical parameter available for comparison is a mixed experimental-theoretical valence angle α(C_2_NH_3_) (Rodler et al., 1986). From a semi-rigid bender analysis of the 9_1,8_-10_0,10_ ground state rotational transition, Rodler and coworkers determined a value of 115.4 ± 0.6° for α(C_2_NH_3_) which is in excellent agreement with our optimized value of 114.76°. To account for vibrational effects **r**_a_ and **r**_g_ parameters have been calculated. While the former correspond to parameters obtained from atomic positions averaged over the VCI ground state wavefunction, the latter are instantaneous inter-nuclear distances calculated from an expectation value of the bond lengths expanded in terms of the normal coordinates. As is typically observed (Czakó et al., 2009; Dinu et al., 2020), both sets of vibrationally averaged bond lengths differ substantially from each other with the largest absolute difference of 0.0169 Å observed for r(NH_3_) in the main isotopologue. The CNH angle α(C_2_NH_3_) shows a slightly larger vibrational effect compared to other angles, in line with the inversion character of this coordinate. The barrier to planarity (C_2*v*_) was computed to be 5249 cm^−1^ at the CCSD(T)-F12b/cc-pVTZ-F12 level and is thus too high for tunneling effects in the fundamental modes to be of any importance. The semi-rigid bender analysis (Rodler et al., 1986) yielded a barrier height of 4700 ± 200 cm-1 which compares well with the present theoretical result. The imaginary frequency characterizing the transition state amounts to i908 cm-1. Note that there is no stationary point on the potential energy surface for a planar structure of neutral ketenimine.

**Table 1:** Computed geometrical parameters of ketenimine and its deuterated isotopologues.

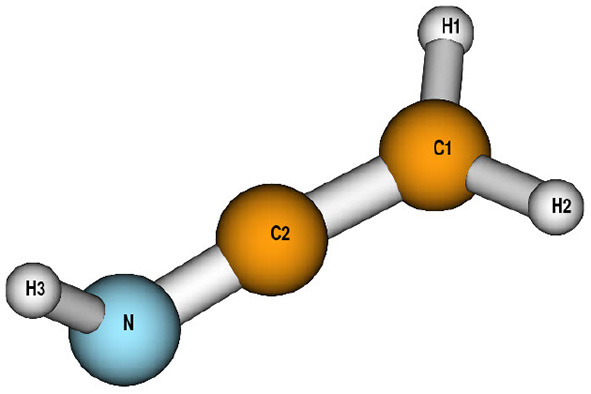			**H**_****2****_**C=C=NH**		**D**_****2****_**C=C=ND**		**H**_****2****_**C=C=ND**	
	**Coord**.	**r_**e**_**	**r_**a**_**	**r_**g**_**	**r_**a**_**	**r_**g**_**	**r_**a**_**	**r_**g**_**
	r(C_1_H_1_)	1.0791	1.0829	1.0991	1.0823	1.0938	1.0818	1.0992
	r(C_1_C_2_)	1.3135	1.3175	1.3205	1.3172	1.3203	1.3173	1.3204
	r(C_2_N)	1.2284	1.2302	1.2335	1.2302	1.2336	1.2303	1.2335
	r(NH_3_)	1.0174	1.0202	1.0371	1.0196	1.0318	1.0211	1.0316
	α(C_2_NH_3_)	114.76	115.07		114.95		115.02	
	α(C_1_C_2_N)	174.05	174.10		174.08		174.10	
	α(H_1_C_1_C_2_)	119.88	119.73		119.78		119.75	

Calculated and experimental (Rodler et al., 1984, 1986) rotational parameters for ketenimine isotopologues are compared in Table 2. There, rotational parameters A, B, and C obtained from the equilibrium geometry are denoted by method **r**^e^. Following the work of Czakó et al. (2009), vibrationally averaged rotational parameters are approximated from the expectation value of the ***μ***-tensor (Watson, 1968) over VCI wavefunctions. An *n*-mode expansion of the ***μ*** -tensor up to 3D terms has been employed in these calculations. Since this approach does not account for Coriolis coupling contributions to the rotational parameters, these are added via a correction based on Vibrational second-order perturbation theory (VPT2) (Rauhut, 2015; Dinu et al., 2020). The final equation for the rotational parameters $B_{v}^{\alpha }$ (α = *a, b, c*) within a vibrational state *v* is thus given by

(3) $$\begin{array}{l} B_{v}^{\alpha }\approx \frac{{\langle \mu_{\alpha \alpha }\rangle }_{v}}{2}+\underset{k}{\sum }\frac{2{\left(B_{e}^{\alpha }\right)}^{2}}{\omega_{k}}\underset{l}{\sum }{\left(\zeta_{kl}^{\alpha }\right)}^{2}\frac{3\omega_{k}^{2}+\omega_{l}^{2}}{\omega_{k}^{2}-\omega_{l}^{2}}\left(v_{k}+\frac{1}{2}\right). \end{array}$$

In Equation (3), 〈μ_αα_〉_*v*_ is the expectation value of a diagonal ***μ***-tensor element evaluated over the VCI wavefunction for state *v*. In the second term of Equation (3), constituting the VPT2 Coriolis correction, $B_{e}^{\alpha }$ is the equilibrium rotational constant with respect to rotation about an axis α, ω_*k*_ are harmonic vibrational frequencies and $\zeta_{kl}^{\alpha }$ are Coriolis constants that describe the coupling of vibrations *k* and *l* via rotation about the α-axis. Results obtained from Equation (3) for the vibrational ground state are denoted either 〈μ_αα_〉_0_ or 〈μ_αα_〉_0_+VPT2 in Table 2, depending on whether the Coriolis correction is included or not. Notice that in the evaluation of 〈μ_αα_〉_*v*_ the ***μ***-tensor has been expanded up to 3D terms.

**Table 2:** Computed and experimental rotational constants in GHz.

**Isotopologue**	**Method**	***A***	***B***	***C***
H_2_C=C=NH	**r**^e^	201.08792 (−0.18%)	9.65878 (−0.05%)	9.47482 (+0.05%)
	〈μ_αα_〉_0_	201.79883 (+0.18%)	9.62993 (−0.34%)	9.43904 (−0.33%)
	〈μ_αα_〉_0_+VPT2	200.31173 (−0.56%)	9.62412 (−0.40%)	9.43309 (−0.39%)
	Exp.^a^	201.44527	9.66315	9.47015
D_2_C=C=ND	**r**^e^	103.66119	8.05874	7.78830
	〈μ_αα_〉_0_	104.74983	8.03709	7.75996
	〈μ_αα_〉_0_+VPT2	103.35526	8.03232	7.75515
H_2_C=C=ND	**r**^e^	162.40310 (−0.51%)	9.03436 (+0.02%)	8.96746 (+0.06%)
	〈μ_αα_〉_0_	163.27301 (+0.02%)	9.00356 (−0.33%)	8.93460 (−0.31%)
	〈μ_αα_〉_0_+VPT2	162.21271 (−0.63%)	8.99879 (−0.38%)	8.92979 (−0.36%)
	Exp.^a^	163.24242	9.03295	8.96219

a *Values determined from fits to Watson's S-reduced Hamiltonian (Rodler et al., 1984, 1986)*.

*Where available, percentage deviations of calculated results with respect to experimental data is given in parentheses*.

Inspection of Table 2 shows rather large deviations of the Coriolis-corrected vibrationally averaged rotational parameters of −0.56, −0.40, and −0.39% with respect to experimental results (Rodler et al., 1984, 1986) for *A*_0_, *B*_0_, and *C*_0_, respectively. In contrast, the calculated equilibrium rotational parameters are in much better agreement with the experimental ground state rotational parameters, which is mainly due to error compensation. To confirm this, a geometry optimization at the all-electron CCSD(T)-F12b level of theory in conjunction with a cc-pCVTZ-F12 basis set (Hill et al., 2010) was carried out. This yields equilibrium rotational parameters (in GHz) for the main ketenimine isotopologue of 202.06657, 9.70127, and 9.51561 for *A*_e_, *B*_e_, and *C*_e_, respectively. Adding the corrections due to vibrational averaging and Coriolis-coupling results in *A*_0_ = 201.290 38 GHz (−0.08%), *B*_0_ = 9.666 61 GHz (+0.04%), and *C*_0_ = 9.464 40 GHz (+0.04%), where deviations with respect to the experimental results of Rodler et al. (1984, 1986) are given in parentheses. The agreement of these corrected results with the experimental ones is excellent, but it is well-known that core correlation effects should not be considered without the inclusion of high-level coupled-cluster terms, e.g., CCSDT(Q), at the same time, because they often partly compensate each other (Ruden et al., 2004; Meier et al., 2011; Puzzarini et al., 2020). Moreover, rovibrational intensities as considered below depend on several quantities and the impact of these additional corrections might be different for the individual quantities. Consequently, there is no unique answer, if the partial inclusion of these corrections will lead to better results. In any case, the inclusion of these high-level corrections is beyond the focus of this study and we neither did account for core correlation effects nor high-order coupled-cluster terms in the calculations presented below.

Experimental high-resolution spectroscopic investigations have revealed strong *a*-axis Coriolis coupling among the low lying vibrational states of ketenimine, especially for the pair of fundamentals ν_8_ and ν_12_ (Bane et al., 2011a,b,c). This can also be shown by comparing rotation-vibration coupling constants $\alpha_{i}^{\beta }$. From the rotational constants presented by Bane et al. (2011a) $\alpha_{i}^{\beta }$ can be approximated by $\alpha_{i}^{\beta }=B_{0}^{\beta }-B_{i}^{\beta }$. This yields 6185.3, −34.0, and -13.4 MHz for $\alpha_{12}^{A}$, $\alpha_{12}^{B}$, and $\alpha_{12}^{C}$, respectively, and −2845.0, −8.1, and -22.1 MHz for $\alpha_{8}^{A}$, $\alpha_{8}^{B}$, and $\alpha_{8}^{C}$, respectively. These values should be compared to our theoretical VPT2 results of 444.5, −34.2, and -14.7 MHz for $\alpha_{12}^{A}$, $\alpha_{12}^{B}$, and $\alpha_{12}^{C}$, respectively, and 2497.5, −9.3 and -22.7 MHz for $\alpha_{8}^{A}$, $\alpha_{8}^{B}$ and $\alpha_{8}^{C}$, respectively. Following Papoušek and Aliev (1982), the latter values have been corrected for the *a*-axis Coriolis resonance between ν_12_ and ν_8_ in order to be comparable with the results of Bane and coworkers. To this end, the corresponding (*i, j*) = (12, 8) or (8, 12) term in the Coriolis contribution to $\alpha_{i}^{\beta }$ (cf. second term in Equation 3) is replaced according to

$$\begin{array}{l} {\left(\zeta_{ij}^{\beta }\right)}^{2}\frac{3\omega_{i}^{2}+\omega_{j}^{2}}{\omega_{i}^{2}-\omega_{j}^{2}}\to -{\left(\zeta_{ij}^{\beta }\right)}^{2}\frac{B_{\text{e}}^{\beta }{\left(\omega_{i}-\omega_{j}\right)}^{2}}{\omega_{i}\omega_{j}\left(\omega_{i}+\omega_{j}\right)}\;. \end{array}$$

We have also investigated whether symmetry allowed off-diagonal contributions $\alpha_{k}^{AC}$ are important for ketenimine, following the work of Aliev and Watson (1985), but found their contribution to effective ${\tilde{\alpha }}_{12}^{\beta }$ and ${\tilde{\alpha }}_{8}^{\beta }$ after diagonalization of the respective $B_{i}^{\alpha \beta }$ matrices negligible.

While the *B* and *C* components are in excellent agreement between experiment and theory, the *A* components show large differences. Moreover, the differences between experiment and theory for ν_12_ and ν_8_ are almost identical but of opposite sign (-5740.8 MHz for ν_12_ and 5342.5 MHz for ν_8_). For comparison, not accounting for Coriolis resonance yields unphysical VPT2 values of −59021.2 and 61996.7 MHz for $\alpha_{8}^{A}$ and $\alpha_{12}^{A}$, respectively. Such effects are unambiguous indications of strong Coriolis coupling. The preceding discussion clearly shows that a simple treatment of the rotational problem and the rovibrational couplings in ketenimine, based on e.g., Equation (3) or VPT2, has to proceed with caution. A variational treatment employing the exact rovibrational Hamiltonian automatically includes all interactions necessary for a correct description of the internal dynamics.

Calculated dipole moments of ketenimine and its isotopologues are listed in Table 3. Our DMS yields equilibrium dipole moments for the main ketenimine isotopologue of ${\;}^{a}\mu_{\text{e}}=\text{0.5008}\;\text{D}$ and ${\;}^{c}\mu_{e}=\text{1.4056}\;\text{D}$, where superscripts *a* and *c* refer to the principal axis components of the dipole vector ${\vec{\mu }}_{\text{e}}$. Symmetric H/D substitution results in a rotation of the *a*- and *c*- axis around the *b* axis. As a consequence, the components ${\;}^{a}\mu_{\text{e}}$ and ${\;}^{c}\mu_{\text{e}}$ of the dipole vector differ among the ketenimine isotopologues but the total dipole moment of $|{\vec{\mu }}_{\text{e}}|=\text{1.4912}\;\text{D}$ is unchanged. The situation is different for the ground state dipole moments ${\vec{\mu }}_{0}$ due to variations of vibrational averaging effects. Overall, vibrational averaging results in a lowering of both *a*- and *c*-axis components. The non-deuterated isotopologue shows slightly larger effects due to vibrational averaging, especially for the *c*-axis component. Rodler et al. (1984) determined the dipole vector components of the main isotopologue and from Stark shifts of the 2_02_←1_01_ and 2_11_←1_10_ rotational transitions. While the resulting ${\;}^{c}\mu_{0}=\text{1.371(6)}\;\text{D}$ is in excellent agreement with our calculated value of 1.3766 D, a somewhat larger difference is observed between the experimental ${\;}^{a}\mu_{0}=\text{0.431(1)}\;\text{D}$ and calculated 0.4587 D. This difference is in part due to a geometric effect. Using the optimized ae-CCSD(T)-F12b/CVTZ-F12 geometry, equilibrium dipole moments of ${\;}^{a}\mu_{\text{e}}=\text{0.4854}\;\text{D}$ (-0.0154 D) and ${\;}^{c}\mu_{\text{e}}=\text{1.4029}\;\text{D}$ (-0.0027 D) were obtained from DCSD/VTZ-F12 calculations, where values in parentheses correspond to the difference with respect to the values quoted in Table 3. Adding the vibrational averaging correction yields an approximate ${\;}^{a}\mu_{0}\approx 0.4433$ D, closer to the experimental result. Again, the influence of high-order coupled-cluster terms would be required to further reduce the remaining error.

**Table 3:** Calculated dipole moments $\vec{\mu }$ (in D) of ketenimine and its deuterated isotopologues.

	${\vec{\mu }}_{\text{e}}$			${\vec{\mu }}_{0}$		
**Isotopologue**	**^a^*μ*_e_**	**^c^*μ*_e_**	**$\left|{\vec{\mu }}_{\text{e}}\right|$**	**^a^*μ*_0_**	**^c^*μ*_0_**	**$\left|{\vec{\mu }}_{0}\right|$**
H_2_C=C=NH^a^	0.5008	1.4056	1.4912	0.4587	1.3766	1.4510
H_2_C=C=ND	0.4643	1.4170	1.4912	0.4314	1.4028	1.4676
D_2_C=C=ND	0.4669	1.4162	1.4912	0.4394	1.3940	1.4616

a *Experimental results (Rodler et al., 1984): ${\;}^{a}\mu_{0}$ = 0.434(1), ${\;}^{c}\mu_{0}$ = 1.371(6), and $\left|{\vec{\mu }}_{0}\right|$ = 1.438(6) D*.

### 3.2. Vibrational Spectrum

The purely vibrational transitions of ketenimine and its isotopologues are listed in Table 4. Clearly, for the deuterated species the majority of experimental assignments is missing and a comparison of the different experimental results for H_2_CCNH shows that these results bear an uncertainty of several wavenumbers.

**Table 4:** Comparison of calculated VCI fundamental frequencies of H_2_C=C=NH and its deuterated isotopologues with experimental data.

		**H**_****2****_**C=C=NH**							**D**_****2****_**C=C=ND**				**H**_****2****_**C=C=ND**			
**#**	**Sym**.	**Harm**.	**VCI**	**Int**.	**Exp.^**a**^**	**Exp.^**b**^**	**Exp.^**c**^**	**Exp.^**d**^**	**Harm**.	**VCI**	**Int**.	**Exp.^**d**^**	**Harm**.	**VCI**	**Int.^**a**^**	**Exp.^**d**^**
ν_1_	A′	3492.7	3315.4	10.9		3321.8			2563.0	2464.4	17.1		3177.1	3046.7	29.7	
ν_2_		3177.0	3048.0	2.6					2325.3	2250.1	40.7	2246	2562.9	2467.4	20.1	
ν_3_		2084.4	2041.8	281.9		2037	2043.6	2040	2044.1	1997.7	150.2	1998	2067.2	2027.7	287.0	2028
ν_4_		1440.3	1435.9	0.1		1355			1231.5	1207.2	0.7		1440.2	1424.7	3.4	
ν_5_		1140.1	1122.5	16.9		1127		1124	944.4	921.7	23.3	921	1136.0	1120.4	11.7	1120
ν_6_		1045.1	1006.7	200.2	1000.2	1004	1000.2	1000	824.5	804.1	77.0	800	829.9	807.7	74.1	817
ν_7_		705.5	691.1	77.2	692.9			690	555.3	549.6	39.8		705.2	679.5	53.4	693
ν_8_		463.3	464.3	19.8	466.5				417.6	415.5	27.0		427.9	426.5	24.0	
ν_9_	A”	3276.2	3132.5	0.2					2441.0	2359.3	0.0		3276.4	3131.4	0.2	
ν_10_		1000.3	980.7	0.6	983.1				842.2	831.0	0.0		1000.4	979.2	0.1	
ν_11_		904.6	876.0	29.6		872		872	666.2	653.7	27.9	648	752.2	731.4	19.1	
ν_12_		405.8	405.7	0.4	409.0				351.4	349.4	0.2		400.8	399.4	0.2	

*Frequencies are given in cm^−1^ and infrared intensities in km/mol*.

a *Experimental gas phase values taken from Bane et al. (2011c,c,a)*.

b *Experimental values taken from the compilation in Bane et al. (2011c)*.

c *Experimental values taken from Ito et al. (1990) and Ito and Nakanaga (2010)*.

d *Experimental Ar matrix values taken from Jacox and Milligan (1963) and Jacox (1979)*.

Concerning the assignments for the non-deuterated ketenimine, a huge difference of more than 80 cm-1 between the computed and experimental values of Bane et al. (2011c) can be seen for mode ν_4_. An analysis showed that this mode shows a strong Fermi resonance with the overtone of ν_7_ and our calculated value of 1350.9 cm-1 for 2ν_7_ agrees nicely with the experimental value of 1355 cm^−1^. As our calculations clearly assign the transition at 1435.9 cm-1 to the fundamental mode, we believe that the experimental value of 1355 cm^−1^ belongs to the 2ν_7_ overtone, which is the lower state of this Fermi pair. The reason for this misassignment might be that the infrared intensity of the overtone is much stronger than that for the fundamental. A closer look at this particular resonance reveals a peculiar feature. While the band intensity at the VSCF level amounts to 3.42 km/mol for ν_4_ and 4.47 km/mol for 2ν_7_, almost all intensity is transferred to the overtone within the VCI calculations. This can be understood by comparison to a VPT2 based analysis that accounts for the Fermi resonance (Vázquez and Stanton, 2007). Then, the intensities of the Fermi dyad in question are predominantly determined by the mixed *a*-axis transition dipole moments ${\langle^{a}\mu \rangle }_{v}$. The latter are obtained from the eigenvector components $C_{\omega }^{\nu }$ of the Fermi resonance matrix and the (deperturbed; dp) transition dipole moments ${\langle^{a}\mu \rangle }_{\omega }^{\text{dp}}$ evaluated over harmonic basis functions |ω〉 according to:

$$\begin{array}{l} {\langle^{a}\mu \rangle }_{\nu_{4}}=C_{\omega_{4}}^{\nu_{4}}\cdot {\langle^{a}\mu \rangle }_{\omega_{4}}^{\text{dp}}+C_{2\omega_{7}}^{\nu_{4}}\cdot {\langle^{a}\mu \rangle }_{2\omega_{7}}^{\text{dp}}\\ \;=0.78\cdot 0.033\text{ D}+0.62\cdot -0.044\text{ D}\approx 0.002\text{ D} \end{array}$$

and

$$\begin{array}{l} {\langle^{a}\mu \rangle }_{2\nu_{7}}=C_{\omega_{4}}^{2\nu_{7}}\cdot {\langle^{a}\mu \rangle }_{\omega_{4}}^{\text{dp}}+C_{2\omega_{7}}^{2\nu_{7}}\cdot {\langle^{a}\mu \rangle }_{2\omega_{7}}^{\text{dp}}\\ \;=-0.62\cdot 0.033\text{ D}+0.78\cdot -0.044\text{ D}\approx -0.055\text{ D}, \end{array}$$

where the corresponding values have been inserted. As can be seen from above equations, the efficient intensity stealing results from a compensation of the signs of the eigenvectors and the deperturbed transition dipole moments. The analysis and composition of all observed resonances of the fundamental modes of all isotopologues based on VCI calculations is summarized in Table 5. For all other fundamental modes of the non-deuterated molecule the agreement of the VCI calculations with the experimental results is excellent and the maximum deviation is no larger than 6.2 cm-1, which is within the error bar of potential energy surfaces obtained from explicitly correlated coupled-cluster theory (Rauhut et al., 2009). In order to reduce this remaining error even further one would need to incorporate a number of corrections within the electronic structure calculations as for example high-order coupled-cluster terms, core-correlation effects, relativistic contributions, etc. (Ruden et al., 2004; Meier et al., 2011).

**Table 5:** Resonances of the fundamental modes of ketenimine and its isotopologues.

**Molecule**	**Mode**	**Freq**.	**Int**.	**Composition**					
H_2_CCNH	ν_4_	1435.9	0.1	4^1^	(55.8%)	7^2^	(35.2%)		
		1350.9	8.4	7^2^	(53.3%)	4^1^	(36.1%)		
	ν_11_	876.0	29.6	11^1^	(59.6%)	8^1^12^1^	(36.7%)		
		880.5	18.6	8^1^12^1^	(57.1%)	11^1^	(39.0%)		
D_2_CCND	ν_4_	1207.2	0.7	4^1^	(69.9%)	6^1^12^1^	(21.4%)		
		1220.6	1.9	6^1^12^1^	(60.3%)	4^1^	(25.6%)		
	ν_3_	1997.7	150.2	3^1^	(49.1%)	4^1^6^1^	(24.8%)		
		2019.2	40.1	4^1^6^1^	(52.3%)	6^2^8^1^	(16.7%)	3^1^	(13.3%)
		2001.0	136.6	6^2^8^1^	(41.2%)	3^1^	(42.0%)		
	ν_1_	2464.4	17.1	1^1^	(65.0%)	6^1^10^2^	(27.5%)		
		2467.0	12.0	6^1^10^2^	(55.2%)	1^1^	(37.2%)		
H_2_CCND	ν_5_	1120.4	11.7	5^1^	(50.9%)	7^1^8^1^	(27.8%)	11^1^12^1^	(16.8%)
		1108.1	8.1	7^1^8^1^	(64.4%)	5^1^	(28.9%)		
		1133.6	4.8	11^1^12^1^	(77.1%)	5^1^	(15.8%)		
	ν_4_	1424.7	3.4	4^1^	(52.7%)	7^2^	(28.5%)		
		1345.3	12.9	7^2^	(57.8%)	4^1^	(31.6%)		

*Frequencies are given in cm^−1^ and infrared intensities in km/mol*.

1, 2 *The superscripts denote the excitation levels of the individual modes*.

The results for the fully deuterated isotopologue, i.e., D_2_CCND are of the same quality as for H_2_CCNH and thus the VCI results most likely provide reliable predictions for all fundamentals. Most remarkably for this isotopologue are the very strong intensities for ν_3_ and its resonance partners (cf. Table 5). The results for H_2_CCND look more inconsistent than for the other two isotopologues. While modes ν_3_ and ν_5_ are in excellent agreement with the experimental results, the VCI results for ν_6_ and ν_7_ deviate by 9.3 and 13.5 cm-1 from the experimental reference data, respectively. According to our VCI calculation, ν_6_ shows a weak Fermi resonance with the overtone of ν_12_ (due to its weak character it has not been listed in Table 5). As such coupling pairs are sensitive with respect to environmental effects as arising from the argon matrix, the deviation of 9.3 cm-1 may be explained this way. However, we consider this rather unlikely, but suspect difficulties in pinpointing the transition energies in the experiment, because Jacox reports that *overlapping parent molecule absorptions and unassigned contributions of other products such as the partially deuterated methyl cyanides complicate the assignment of other absorptions to the partially deuterated ketenimines* (Jacox, 1979). Note that for all isotopologues, ν_4_ shows strong Fermi resonances, but with different partners and in all cases the intensity of the overtone is stronger than for the fundamental.

### 3.3. Rotational Spectrum

**Figure 1:**
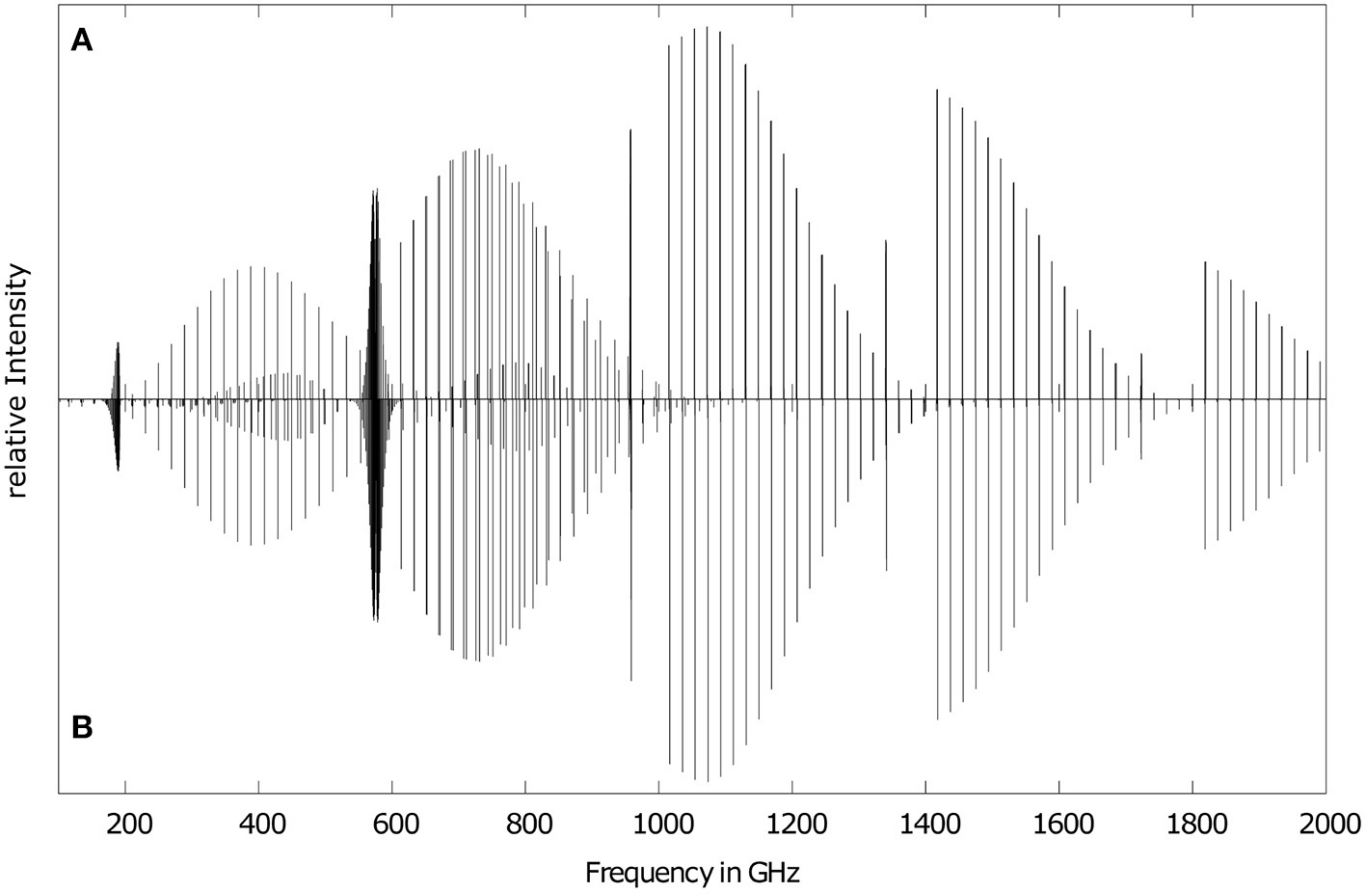
Rotational spectrum of ketenimine in its ground vibrational state for *T* = 50 K. Comparison of the theoretical RVCI results **(A)** obtained from Molpro and **(B)** based on experimental results as determined by Degli Esposti et al. (2014) (see text for details).

In Figure 1, our computed RVCI rotational spectrum of ketenimine (Figure 1A) is compared with a simulated experimental spectrum (Figure 1B) for a temperature of *T* = 50 K. The latter spectrum has been calculated with the Spcat program (Pickett, 1991) using the spectroscopic parameters of Degli Esposti et al. (2014) determined from the submillimeter wave spectrum, while intensities are based on the experimentally determined dipole moments in the vibrational ground state (Rodler et al., 1984). Intensities are given relative to the strongest line at *T* = 50 K, which corresponds to the 5_2,3_ → 6_3,3_ transition in both cases. Excellent agreement between the RVCI and the experimental spectrum is observed. Only very subtle frequency differences appear upon close inspection, which occur mainly due to the difference of about 0.3 GHz in the employed *A* rotational constants (compare Table 2). The overall shape of the spectrum, which is dominated by ${\;}^{c}R_{10}$ branch progressions and the sharp ^*c*^*Q* branches, is nicely reproduced by the RVCI spectrum.

**Figure 2:**
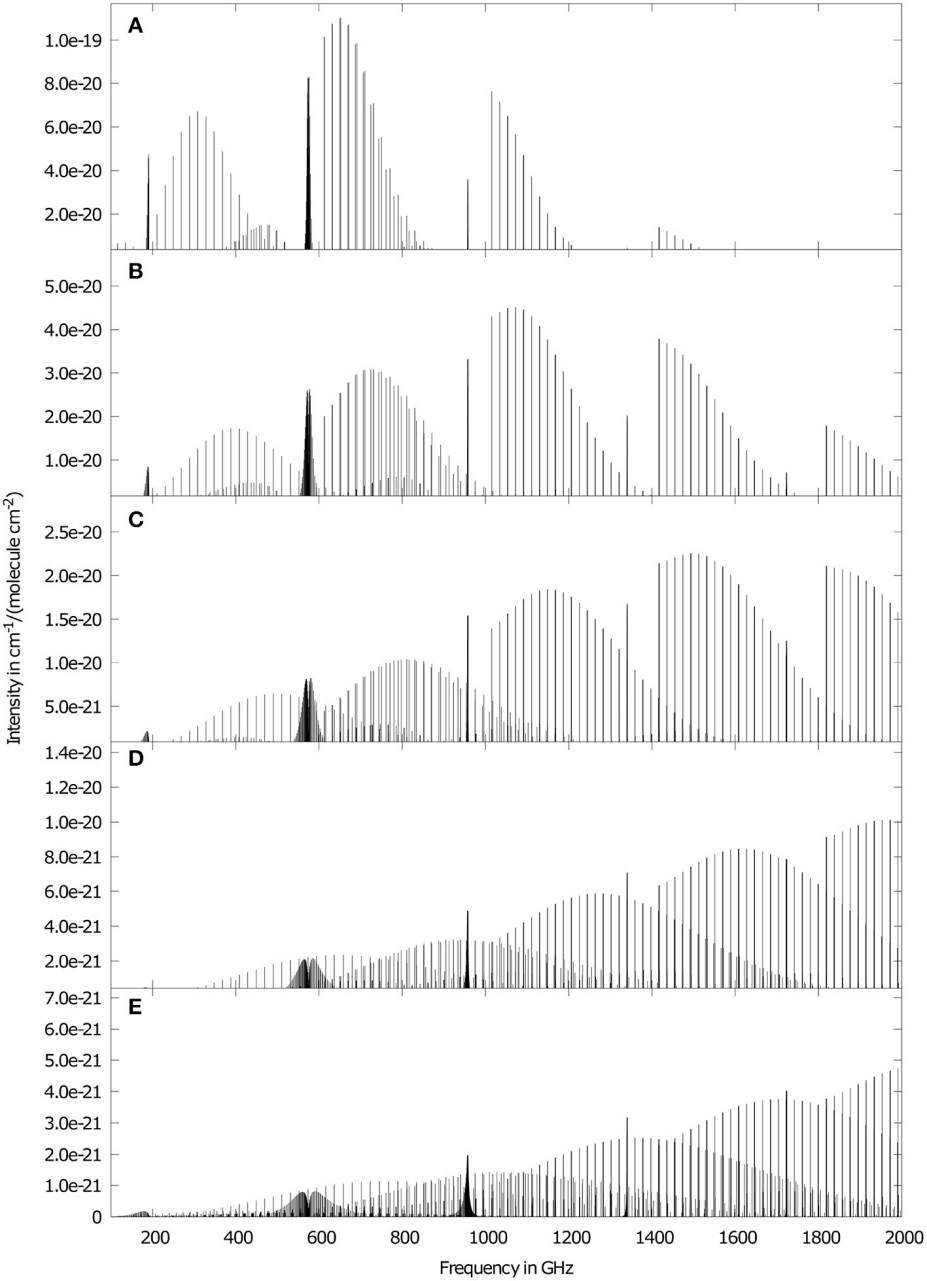
Ground state rotational RVCI spectrum of ketenimine for different temperatures, **(A)** 20 K, **(B)** 50 K, **(C)** 100 K, **(D)** 200 K, **(E)** 300 K. Temperatures are considered only in the occupation numbers and not in line broadening. For the sake of clarity, the intensity axis of adjacent sub-figures are downscaled by a factor of two for increasing temperatures.

We have studied the temperature dependence of the ground state rotational spectrum in the range 20 to 300 K and results are depicted in Figure 2. With increasing temperature the intensity of the rotational transitions decrease by about a factor of 2 and the rather sharp ${\;}^{c}Q_{10}$ branches below 600 GHz broaden significantly. While for *T* = 20 K the ${\;}^{c}R_{10}$ transitions originating in *K*_*a*_ = 1 states are the strongest up to 2 THz, with each increase in temperature the maximum shifts by one unit in *K*_*a*_. Furthermore, the effect of asymmetry splitting in *K*_*a*_ = 1 states are clearly visible. All these observations originate in the Boltzmann distribution function resulting in a higher partition function and a shift in the thermal distribution toward higher *K*_*a*_ and *J*. The former is responsible for the emergence of higher energy branches and the latter for the shifts in the maximum for individual *J*-progressions, highlighting the importance of an accurate determination of the partition function.

### 3.4. Rovibrational Spectrum

**Figure 3:**
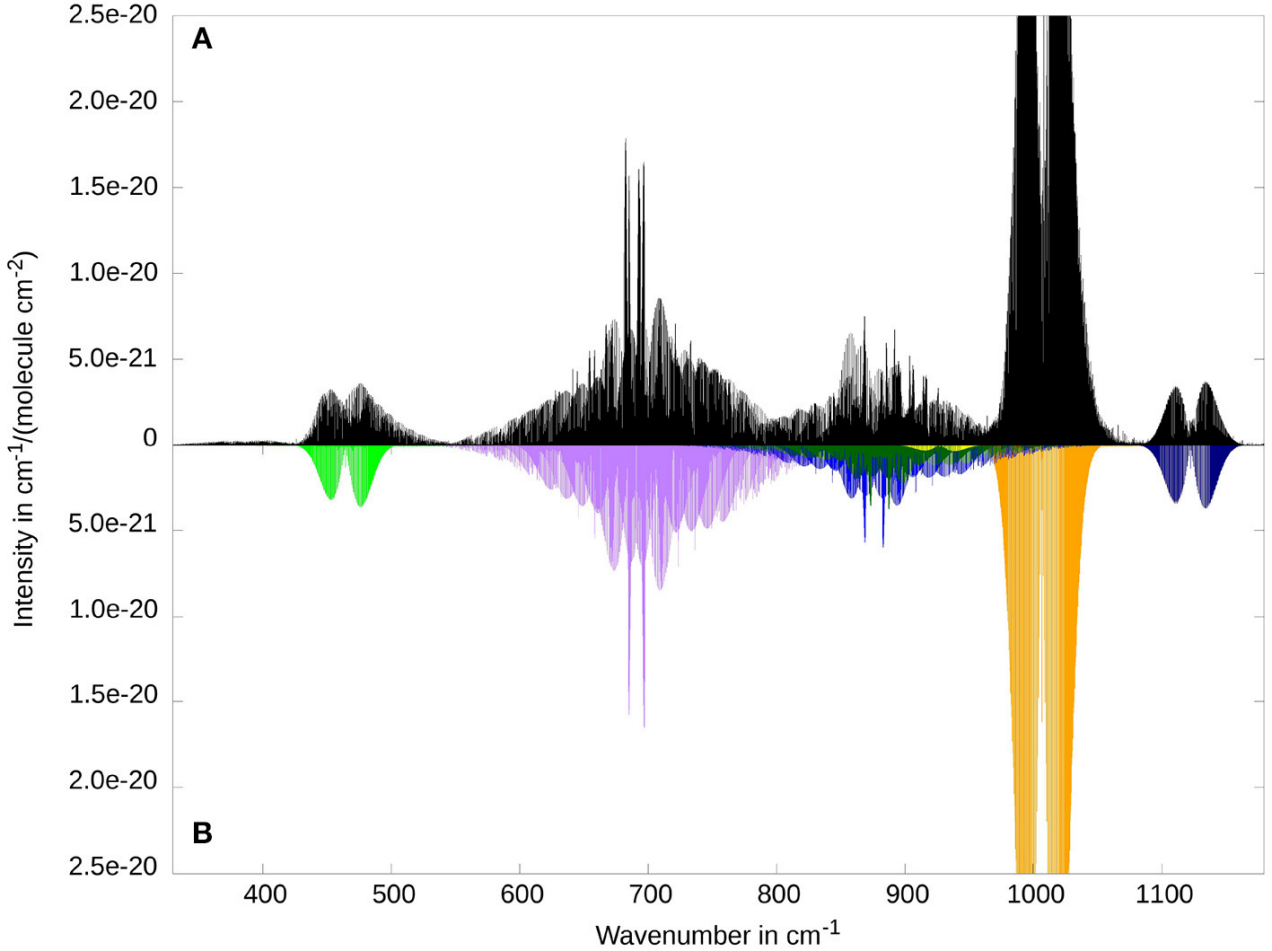
Overview of the low frequency range of the rovibrational spectrum of ketenimine. Comparison between RVCI **(A)** and RCI **(B)** results. Visible contributions are provided by the fundamental bands ν_8_ (at 464.4 cm^−1^, CCN in-plane bend, in light green), ν_7_ (at 691.2 cm^−1^, CH_2_ wagging, in purple), ν_11_ (at 876.2 cm^−1^, torsion, in light blue), ν_6_ (at 1007.1 cm^−1^, CNH bend, in orange), ν_5_ (at 1122.5 cm^−1^, CCN stretch, in dark blue) as well as the combination band ν_8_ + ν_12_ (at 880.7 cm^−1^ in dark green), and the overtone 2ν_8_ (at 927.3 cm^−1^ in yellow).

**Figure 4:**
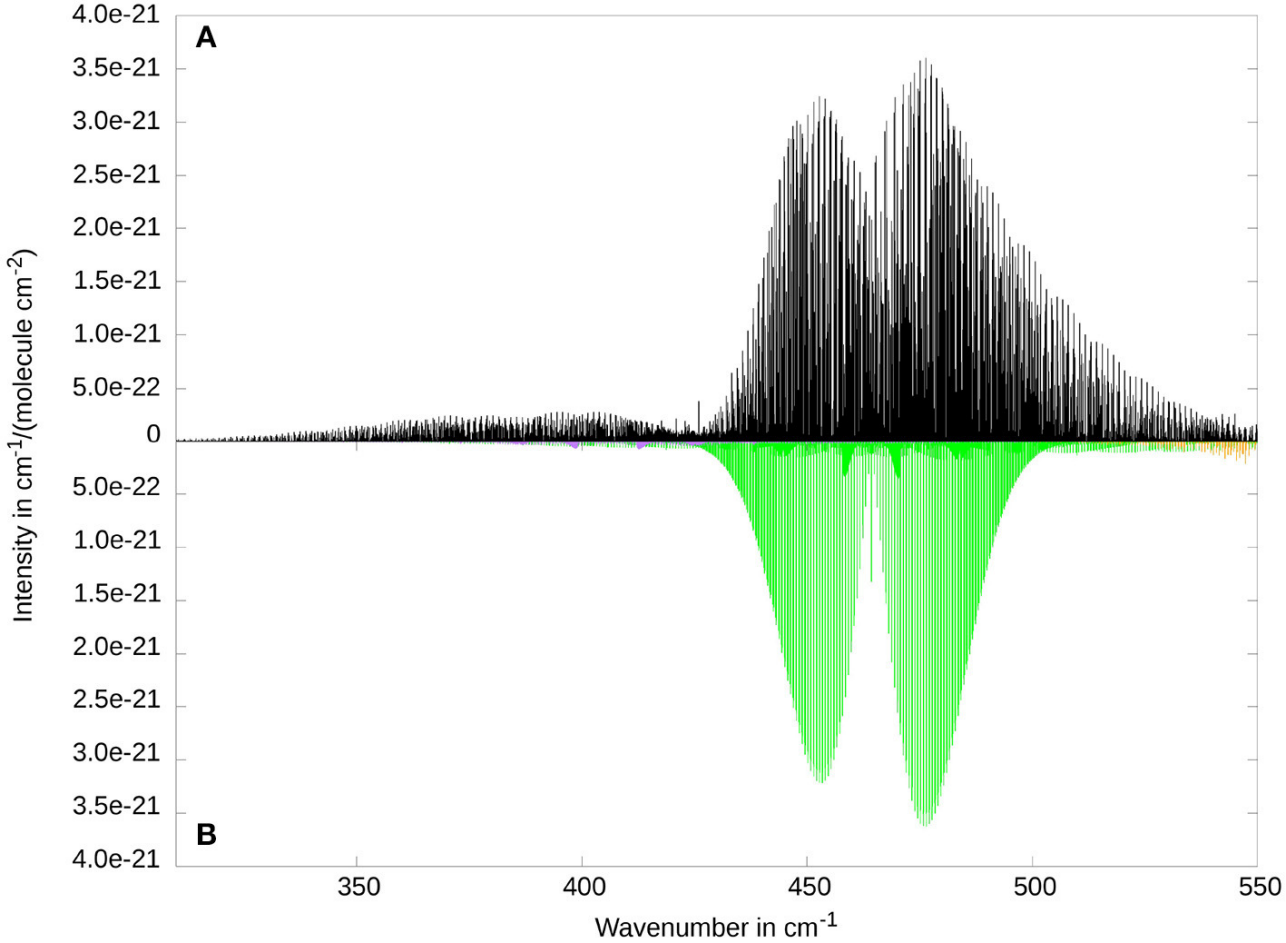
The two lowest fundamental bands ν_12_ (at 405.7 cm^−1^, CCN out-of-plane bend, VCI intensity 0.4 km/mol, in purple) and ν_8_ (at 464.4 cm^−1^, CCN in-plane bend, VCI intensity 19.8 km/mol, in light green) as well as small contributions of ν_7_ (at 691.2 cm^−1^, CH_2_ wagging, in orange). Comparison between RVCI **(A)** and RCI **(B)**.

**Figure 5:**
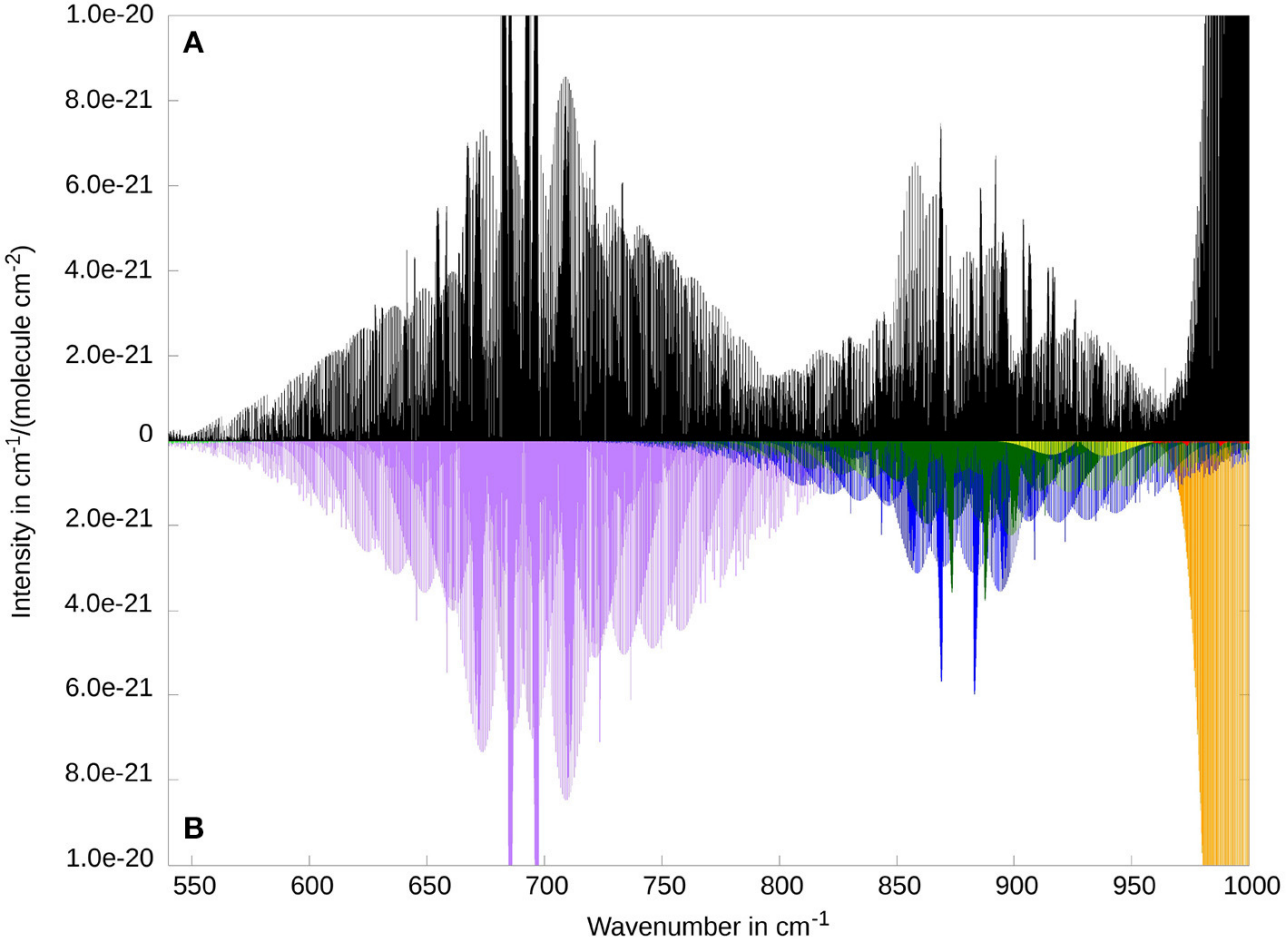
Rovibrational spectrum of ketenimine of the fundamental bands ν_7_ (at 691.2 cm^−1^, in purple), ν_11_ (at 876.2 cm^−1^, in light blue), ν_10_ (at 980.7 cm^−1^, in red, barely visible) and ν_6_ (at 1007.1 cm^−1^ in orange) and the combination band ν_8_ + ν_12_ (at 880.7 cm^−1^ in dark green), and the overtone 2ν_8_ (at 927.3 cm^−1^ in yellow). Comparison between RVCI **(A)** and RCI **(B)** results.

It is known that the rovibrational spectrum of ketenimine shows many strongly coupled rovibrational bands in the energy regime between 300 and 1200 cm^−1^ (Bane et al., 2011a,b,c). For this reason, we want to give a broad overview over this area with Figure 3. The figure shows the 5 fundamental bands ν_5_, ν_6_, ν_7_, ν_8_, ν_11_, as well as the overtone 2ν_8_ and the combination band ν_12_ + ν_8_. The comparison between RVCI and RCI spectra allows for a better understanding of the coupling and resonance effects. Two examples for these couplings can be seen around 400 and 900 cm-1. Therefore these areas are displayed in separate Figures 4 and 5 and will be discussed below. For the following figures, we did not use any line broadening, since no direct comparison with experimental results is depicted.

A good example for the strong Coriolis-coupling in this system can be seen in Figure 4 between ν_8_ and ν_12_. The bottom subplot (Figure 4B) shows the results of the RCI calculation, where Coriolis-coupling is not considered. ν_12_ has about two orders of magnitude less intensity then ν_8_, in line with the band intensities obtained from VCI (cf. Table 4). The in-plane CCN bending vibration ν_8_ clearly shows an *A*-type spectrum, whereas the out-of-plane CCN bend ν_12_ shows the expected *B*-type intensity pattern. Inclusion of Coriolis-coupling in the upper subplot (Figure 4A) results in ν_12_ gaining about one order of magnitude in intensity by intensity borrowing in the RVCI calculation. The missing intensity in the stronger band is hard to see, since the relative difference is smaller. Furthermore, ν_12_ can neither be assigned to an *A*-type nor a *B*-type band structure, due to the lifting of the typical selection rules via Coriolis-coupling. For the ν_8_ fundamental, the overall shape of an *A*-type transition is retained. Both bands show a rather asymmetric structure, with a supposed band center of the ν_12_ mode shifted by about 20 cm-1 to lower energies and visible transitions well below 350 cm-1. This is in contrast to ν_8_, where both branches gain intensity toward higher energies, with an overlapping region at about 470 cm-1. The high energy tails of the *R*-branch (around 530 cm-1) could also be influenced by the ν_7_ mode (orange in Figure 4). However, we expect that effect to be small, since the VCI energies of the two modes ν_7_ and ν_8_ are separated by more than 200 cm-1.

A comparison with the experimental and simulated spectra of Bane and coworkers (Figures 2A and B in Bane et al., 2011c) shows in general good agreement for ν_12_ (Figure 4 as well as in Supplementary Figure 1). This holds for both the number of progressions and their distribution over the spectral range from 330 to 410 cm-1. However, there seems to be a sudden drop in intensity at 410 cm-1 that can not be found in our calculated results. The slight shift of our RVCI-spectrum by about 4 cm-1 compared to experiment can be explained by our error in the VCI energy of 3.3 cm-1 (cf. Table 4). Comparing the spectra of Bane et al. for ν_8_ (Figures 2C and D in Bane et al., 2011c) with ours (compare also in Supplementary Figure 2) shows somewhat larger deviations. While the *A*-type *P* and *R* branch structure is still recognizable in Figure 4, the spectra of Bane et al. show a broader distribution of the *K*_*a*_ sub-bands leading to the *A*-type band shape being partially obscured. It should be mentioned that there are isolated peaks protruding both bands (see Bane et al., 2011c). Tests have shown (see Supplementary Figures 1–4) that such prominent peaks as well as the above discussed differences in the intensity patterns originate from line broadening. Since we do not use any broadening, those protruding peaks cannot be expected in our spectrum, but of course must appear in the experimental spectra.

In contrast to the previously considered modes, the CH_2_ wagging mode ν_7_ does not change its macroscopic shape due to Coriolis coupling. As can be seen in Figure 5, the general form of ν_7_ corresponds to a *C*-type transition of a near-prolate asymmetric top molecule. The main difference due to RVCI (in the top panel) is the splitting of the central *Q*-branches. In comparison with the work of Bane et al. (Figure 2B in Bane et al., 2011b), there are two small deviations besides the overall good agreement (compare also in Supplementary Figure 3). First, there is a small shift for the two high peaks in the middle of the mode. Second, the experiment seems to show a sudden drop in intensity between the middle (650 and 730 cm-1) and the outer parts of the progression (above 730 and 650 cm-1). As mentioned before, the distributed peaks shown in the paper of Bane et al. (2011b), are caused by a Gauss broadening of the experimental results and are therefore not to be expected in our spectra. The possible coupling of ν_7_ with higher energy modes (above 750 cm-1 in Figure 5) is not shown in the simulated spectrum of Bane et al. (2011b).

Another example of extensive rovibrational coupling occurs between 780 and 970 cm-1 (see Figure 5). The reason for this is the close proximity of three vibrational bands: one fundamental band (ν_11_ at 876.2 cm-1, torsion, *A*″ symmetry), one combination band (ν_8_ + ν_12_ at 880.7 cm-1, *A*″ symmetry) and one overtone (2ν_8_ at 927.3 cm-1, *A*′ symmetry) within 50 cm-1. Additionally, there is a further “dark state” involved, corresponding to the overtone of the out-of-plane CCN bending mode (2ν_8_ at 809.6 cm-1, *A*′ symmetry). While the overtones 2ν_12_ and 2ν_8_ are strongly coupled to the combination band ν_8_ + ν_12_ via *a*-axis Coriolis-coupling ($\zeta_{8,12}^{a}=-0.802$), similar to the correspondingly coupled fundamentals, the ν_11_ fundamental has been shown to be in Fermi resonance with the combination band (cf. Table 5). The resulting rovibrational coupling leads to an almost complete loss of discernible band structure when comparing the RCI (bottom panel) and the RVCI spectrum (top panel). As a consequence, experimental assignment and interpretation of this spectral region will be highly difficult without reliable estimates of spectroscopic parameters obtained from theory.

**Figure 6:**
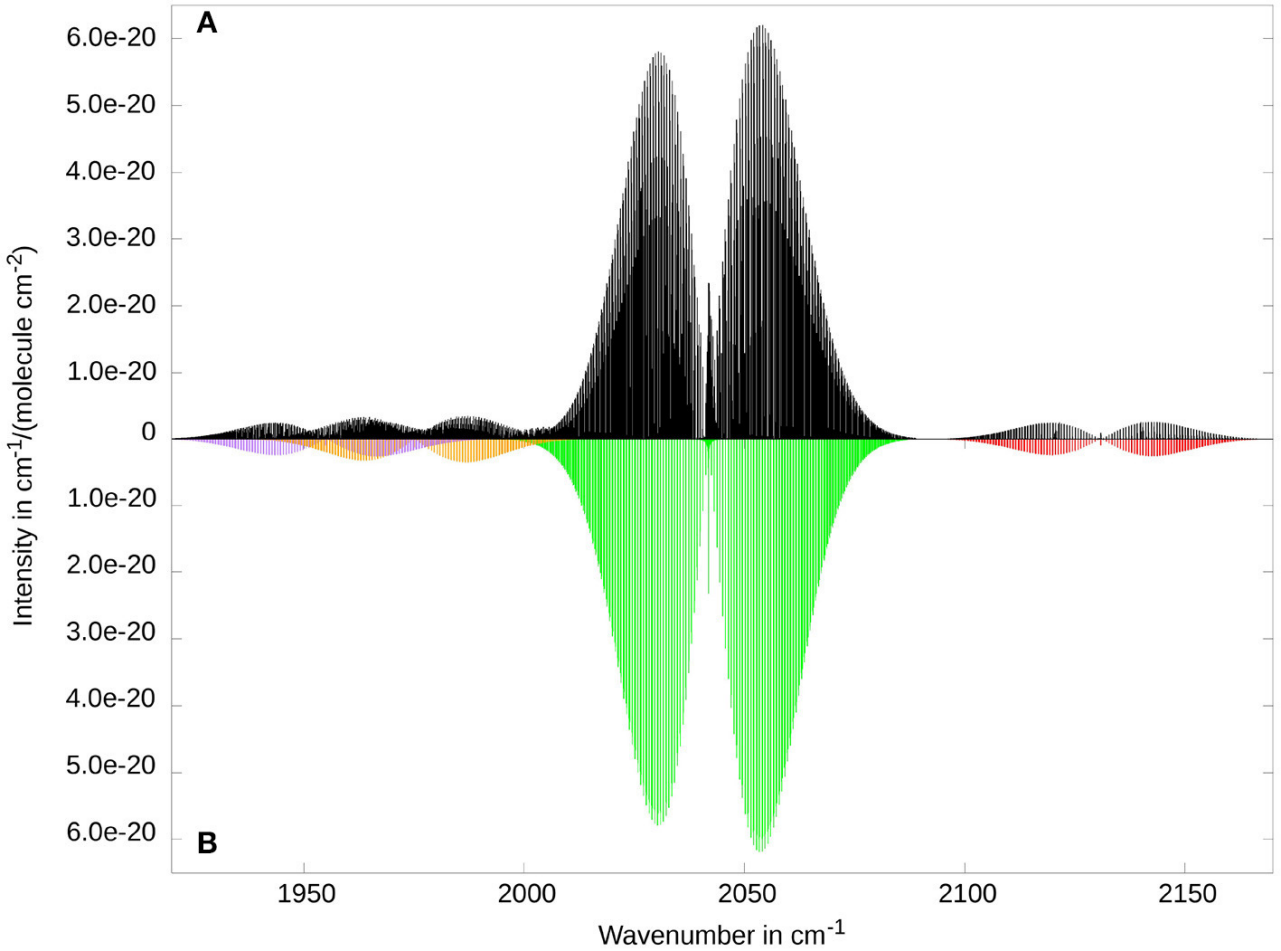
Rovibrational spectrum of ketenimine in the region of the strongest fundamental band ν_3_ (at 2041.8 cm^−1^, CCN stretch, in green). Additionally, the overtones 2ν_10_ (at 1955.0 cm^−1^, in purple), and 2ν_6_ (at 1975.1 cm^−1^, in orange), as well as the combination band ν_5_ + ν_6_ (at 2130.8 cm^−1^, in red) provide visible contributions in this spectral range. Comparison between RVCI **(A)** and RCI **(B)** results.

Figure 6 reveals only very weak Coriolis coupling between ν_3_ and 2ν_6_, respectively 2ν_10_. One reason for this is that the largest non-vanishing ζ constants for $\zeta_{3,6}^{\alpha }$ and $\zeta_{3,10}^{\alpha }$ correspond to *b* and *c* direction, respectively. Hence the rotational constants along the *b* and *c* direction have to be considered. Since they are a factor of 20 smaller then the *A* rotational constant, the coupling is significantly weaker. In addition to that, Coriolis coupling between a fundamental band and the overtone of another band requires at least the first order in the ***μ***-tensor expansion. Therefore, it is possible that in experiments a somewhat stronger coupling occurs, even though it is unlikely due to the small rotational constants. The only experimental results for this mode have been presented by Ito et al. (1990). While a direct comparison of spectra is ambiguous, due to relatively low resolution and a contamination of the experimental probe, Ito et al. do note signs of Coriolis perturbations in the fitted effective spectroscopic parameters of the ν_3_ band.

**Figure 7:**
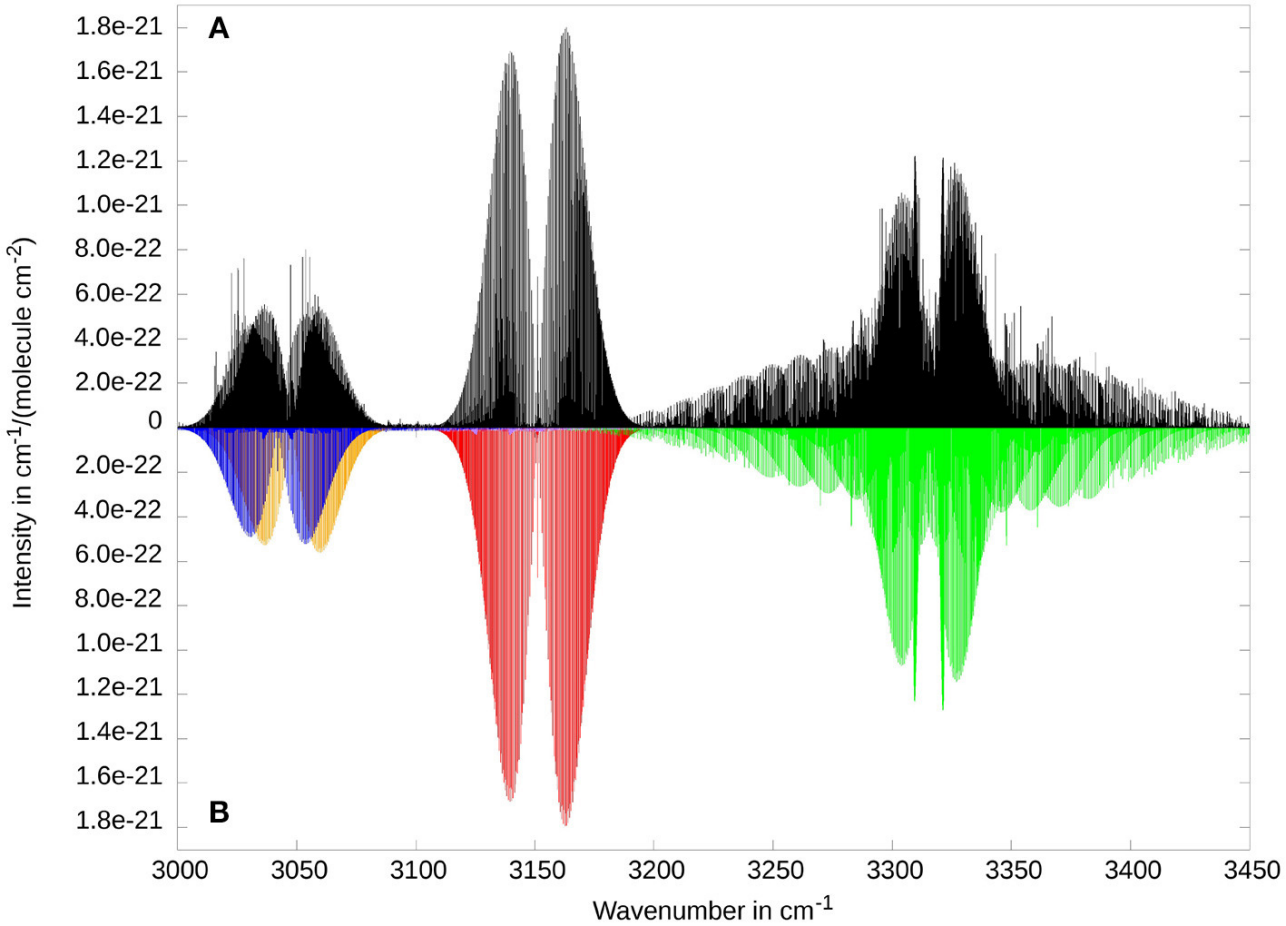
Rovibrational spectrum of ketenimine in the region of XH stretching fundamentals. Three fundamental bands ν_2_ (at 3048.0 cm^−1^, symmetric CH_2_ stretch, in orange), ν_9_ (at 3132.5 cm^−1^, anti-symmetric CH_2_ stretch, in purple, barely visible) and ν_1_ (at 3315.4 cm^−1^, NH stretch, in light green) are shown, as well as the combination bands ν_3_ + ν_5_ (at 3042.0 cm^−1^, in blue) and ν_3_ + ν_6_ (at 3151.1 cm^−1^in red). Comparison between RVCI **(A)** and RCI **(B)** results.

Figure 7 shows the XH stretching fundamental region between 3000 and 3500 cm^−1^. The corresponding fundamental bands are the symmetric (ν_2_) and antisymmetric (ν_9_) CH_2_ stretch vibrations and the NH stretching mode (ν_1_), in energetically ascending order. Additionally, the ν_3_ + ν_5_ and the ν_3_ + ν_6_ combination bands provide a significant contribution to the spectrum. All bands show the expected shapes of *A*-type (ν_2_, ν_3_ + ν_5_, and ν_3_ + ν_6_), *B*-type (ν_1_), and *C*-type (ν_9_) transitions. So far there were no experimental results published for any of these bands. The comparison between RCI (bottom) and RVCI results (top) gives no indications for substantial Coriolis coupling among the fundamental bands. This is supported by taking the corresponding Coriolis coupling constants into account, where the largest (absolute) value is found for $\zeta_{2,9}^{c}\approx 0.05$. Due to the restriction to a constant ***μ***-tensor, no direct Coriolis coupling between fundamentals ν_*i*_ and combination bands ν_*j*_ + ν_*k*_ is included in the RVCI-matrix. While a strong interaction between ν_2_ and ν_3_ + ν_5_ is unlikely because of very small ζ-constants ($|\zeta_{2j}^{b}|\approx 0.003$), such a coupling might be relevant for the ν_9_ fundamental due to the close by ν_3_ + ν_6_ combination band and the substantial intensity difference. However, experimental observation of ν_9_ will be complicated by the fact that ν_9_ rovibrational transitions will most likely be hidden in between the stronger ν_3_ + ν_6_ band. Overall the rovibrational transitions in this spectral region have rather low intensity compared to other spectral regions. The VCI band intensity of the strongest vibrational transition ν_1_ (10.9 km/mol) is already a factor of about 20 lower than the two strongest fundamental bands (cf. Table 4).

### 3.5. Summary and Conclusions

The vibrational, rotational and rovibrational spectra of ketenimine have been studied by high-level *ab initio* methods for the first time. Based on a new series of almost black-box algorithms being implemented in the Molpro package of quantum chemical programs, it was possible to simulate and analyze the complex rovibrational features of this near-prolate asymmetric top molecule. Note, that the input information for these calculations comprises just the molecular structure and the call of the requested modules, which act in a highly optimized—with respect to memory requirements and CPU time—and automated manner. Agreement with available experimental data, i.e., ground state rotational constants, vibrational band origins, dipole moments or the rotational spectrum as a whole, was found to be excellent or at least very good. Beside the reliable reproduction of experimental reference data, many predictions could be provided, which we consider a trustworthy guidance for new experimental studies or astrochemical observations. The occurrence of several Fermi resonances even for fairly low lying transitions requested accurate potential energy and dipole surfaces, which has been accomplished by explicitly correlated coupled-cluster theory and the rather new distinguishable clusters approximation. A proper description of these resonances was found to be important for the subsequent rovibrational calculations. For example, the strong Fermi resonance of mode ν_11_ with the combination band ν_8_ + ν_12_ has significant impact on the spectrum, but was not discussed in the experimental work (Bane et al., 2011a). This example demonstrates the benefits, that can arise from combined experimental and theoretical studies to provide reliable reference data for astrophysical studies. Covering a wide spectral range and identifying signature areas within the spectrum are challenging goals in the future. Currently, work is in progress to include coupling terms originating from higher-order ***μ***-tensor terms and hot bands.

## Data Availability Statement

The original contributions generated for the study are included in the article/Supplementary Material, further inquiries can be directed to the corresponding author/s.

## Author Contributions

MT performed all RVCI calculations, generated all figures, and wrote parts of the text. GR supervised the project, performed the VCI calculations, and wrote parts of the text. SE wrote large parts of the program being used for producing the data. BS performed VCI and RCI calculations, analyzed the spectra, and wrote parts of the text. All authors contributed to the article and approved the submitted version.

## Conflict of Interest

The authors declare that the research was conducted in the absence of any commercial or financial relationships that could be construed as a potential conflict of interest.
