# Supplementary material for: High-Level Rovibrational Calculations on Ketenimine
Source: Front Chem. 2021 Jan 6;8:623641. doi: 10.3389/fchem.2020.623641 (PMC7873934; doi:10.3389/fchem.2020.623641)
Supplement: Supplementary file 1 [file Data_Sheet_1.PDF]

Supporting information on:

High-level rovibrational calculations on ketenimine

Martin Tschöpe, Benjamin Schröder, Sebastian Erfort, Guntram Rauhut*

Institute for Theoretical Chemistry, University of Stuttgart, Pfaffenwaldring 55, 70569 Stuttgart, Germany.
E-mail: rauhut@theochem.uni-stuttgart.de

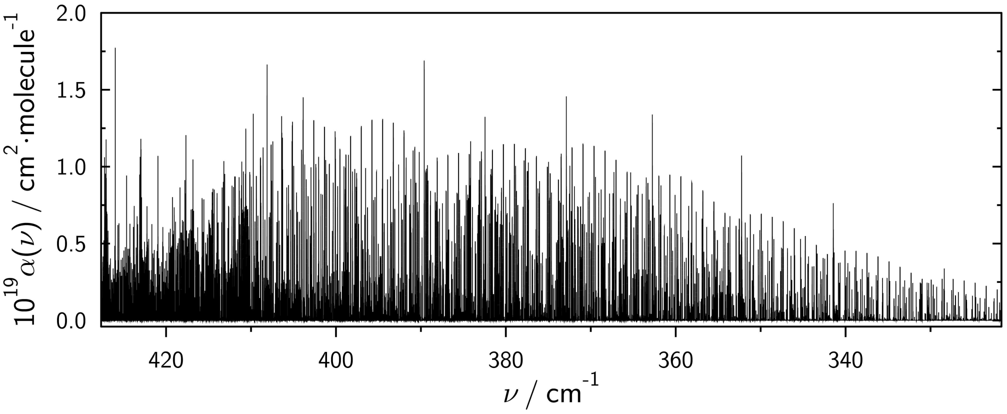

Figure S1: Calculated rovibrational spectrum of the ν_{12} band in ketenimine ($H_2C{=}C{=}NH$) at 300 K. The spectrum is obtained by convolving the calculated line intensities with a gaussian profile (FWHM = 0.002 cm).

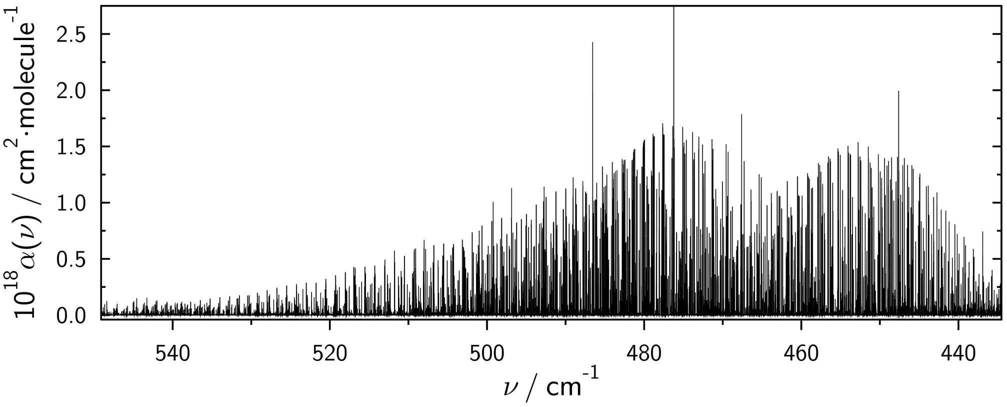

Figure S2: Calculated rovibrational spectrum of the ν_8 band in ketenimine ($H_2C{=}C{=}NH$) at 300 K. The spectrum is obtained by convolving the calculated line intensities with a gaussian profile (FWHM = 0.002 cm^{-1}).

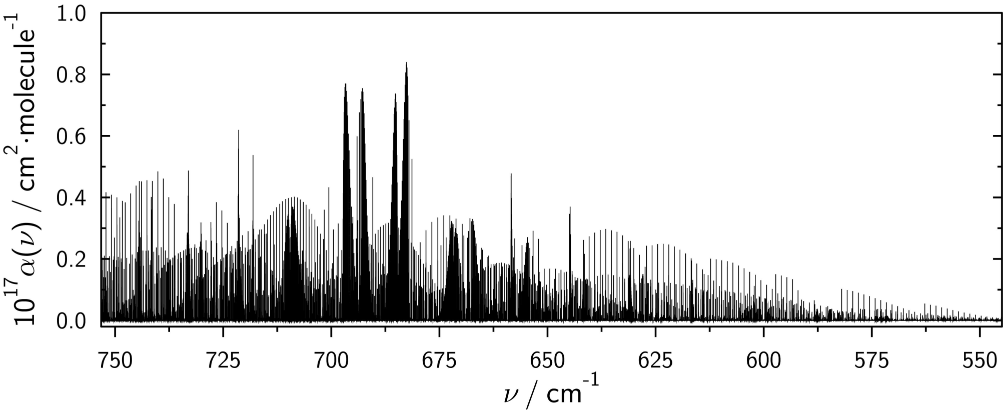

Figure S3: Calculated rovibrational spectrum of the ν_7 band in ketenimine ($H_2C{=}C{=}NH$) at 300 K. The spectrum is obtained by convolving the calculated line intensities with a gaussian profile (FWHM = $0.002\,\mathrm{cm}^{-1}$).

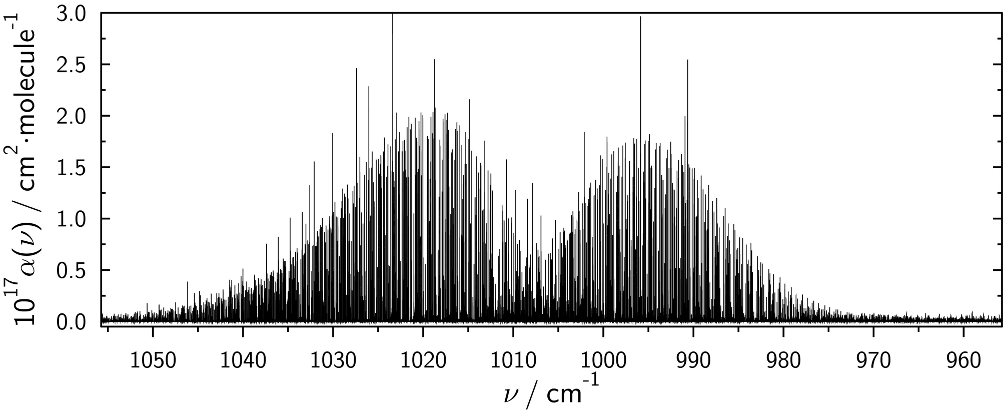

Figure S4: Calculated rovibrational spectrum of the ν_6 band in ketenimine ($H_2C{=}C{=}NH$) at 300 K. The spectrum is obtained by convolving the calculated line intensities with a gaussian profile (FWHM = $0.002\,\mathrm{cm}^{-1}$).
